# The Burden of a Remote Trial in a Nursing Home Setting: Qualitative Study

**DOI:** 10.2196/jmir.9638

**Published:** 2018-06-19

**Authors:** Susie Donnelly, Brenda Reginatto, Oisin Kearns, Marie Mc Carthy, Bill Byrom, Willie Muehlhausen, Brian Caulfield

**Affiliations:** ^1^ Applied Research for Connected Health University College Dublin Dublin Ireland; ^2^ ICON plc Dublin Ireland

**Keywords:** mHealth, patient burden, remote trial, clinical trial methodology, wearable technology, activity monitors

## Abstract

**Background:**

Despite an aging population, older adults are typically underrecruited in clinical trials, often because of the perceived burden associated with participation, particularly travel associated with clinic visits. Conducting a clinical trial remotely presents an opportunity to leverage mobile and wearable technologies to bring the research to the patient. However, the burden associated with shifting clinical research to a remote site requires exploration. While a remote trial may reduce patient burden, the extent to which this shifts burden on the other stakeholders needs to be investigated.

**Objective:**

The aim of this study was to explore the burden associated with a remote trial in a nursing home setting on both staff and residents.

**Methods:**

Using results from a grounded analysis of qualitative data, this study explored and characterized the burden associated with a remote trial conducted in a nursing home in Dublin, Ireland. A total of 11 residents were recruited to participate in this trial (mean age: 80 years; age range: 67-93 years). To support research activities, we also recruited 10 nursing home staff members, including health care assistants, an activities co-ordinator, and senior nurses. This study captured the lived experience of this remote trial among staff and residents and explored the burden associated with participation. At the end of the trial, a total of 6 residents and 8 members of staff participated in semistructured interviews (n=14). They reviewed clinical data generated by mobile and wearable devices and reflected upon their trial-related experiences.

**Results:**

Staff reported extensive burden in fulfilling their roles and responsibilities to support activities of the trial. Among staff, we found eight key characteristics of burden: (1) comprehension, (2) time, (3) communication, (4) emotional load, (5) cognitive load, (6) research engagement, (7) logistical burden, and (8) product accountability. Residents reported comparatively less burden. Among residents, we found only four key characteristics of burden: (1) comprehension, (2) adherence, (3) emotional load, and (4) personal space.

**Conclusions:**

A remote trial in a nursing home setting can minimize the burden on residents and enable inclusive participation. However, it arguably creates additional burden on staff, particularly where they have a role to play in locally supporting and maintaining technology as part of data collection. Future research should examine how to measure and minimize the burden associated with data collection in remote trials.

## Introduction

Older adults, particularly those with impaired mobility and cognitive disorders such as dementia, are typically underrecruited in clinical trials, even though they have the greatest need for health care services [1-6]. Reasons for underrepresentation are unclear, but may relate to comorbidities, communication difficulties (due to hearing or vision impairments), and physical immobility that constrains transportation to a research site [7-9]. The perception among health care providers that the clinical research experience is stressful or burdensome can mean that researchers elect not to access patients [10]. Although remote clinical trials may resolve the burden associated with clinical trial participation and make clinical research participation more accessible for traditionally hard-to-reach groups, this has been largely unexplored, particularly in terms of understanding the role of mobile and wearable technology for supporting a partially or wholly remote trial.

Historically, efforts to ascertain and address the burden associated with clinical research have tended to focus on direct risks of clinical interventions or data collection procedures, such as survey participation [11-13]. Lingler et al argued that a complete conceptualization of research burden encompasses not only perceptions regarding direct risks involved in the study but also the indirect burdens [14]. These indirect burdens may to vary based on the duration of research, intensity, and invasiveness of study procedures. Moreover, burden may be modulated by the perceived benefit of participation [14]; in other words, the value of participation. Ulrich et al suggested that although institutional review boards regularly evaluate risks and benefits of research on human subjects, how patients perceive burden and benefits of clinical trial participation remains to be extensively and qualitatively examined [15].

As yet, there is no clear and consistent definition of a remote trial in the existing literature. Rather, there are a range of interchangeable descriptive terms ranging from “web-based,” “virtual,” and “nonclinical” trials, to “place-shifted” and “remotely-monitored” trials. Fundamentally, a remote trial follows the underlying principle of placing individuals, rather than investigative sites, at the center of the research process using mobile and wearable technology to support data collection. Covington and Veley claim that a remote patient-centered model offers great potential to advance observational studies and randomized controlled trials [16]. It also offers an opportunity to increase research efficiency through remote patient recruitment and enrollment, retention programs, data collection, and long-term follow-up evaluation. At the same time, this operational approach intensifies patient-centeredness by directly engaging patients in research, overcoming geographic obstacles to connect stakeholders, and incorporating patient input into the research process [16].

There is evidence that some clinical trials have leveraged digital technology to varying extents [16-21]. A study published in 2014 involving researchers from Pfizer marked a clear departure from the traditional clinical model. It sought to purposefully design and conduct a “virtual” trial. However, recruitment was problematic and of the 5157 individuals who registered on the trial website, only 18 were randomized to treat [20]. Although the online informed consent process was successful, for those patients progressing to that stage, the lack of human interaction in the recruitment process was a major barrier (particularly in population studies that predominantly comprise of older adult subjects) [22,23]. This study demonstrated the need for understanding potential sources of burden in a remote trial. It is proposed that the application of mobile and wearable technologies may help in innovating clinical trials toward a more patient-centered approach. However, this shift in location must consider the unintended consequences, namely, the relevant stakeholders who potentially absorb the burden associated with clinical trial participation. When targeting older adults with impaired mobility and cognitive disorders, such stakeholders may include family members, caregivers, or nursing home staff. Thus, to determine the feasibility of a remote trial, it is essential to understand the burden associated with remote data collection on the stakeholders. As our population ages, nursing homes and residential care settings are increasingly important study sites. Thus, in this study, we aimed to explore the burden associated with a remote trial in a nursing home for staff and residents.

## Methods

### Remote Trial

Between March 2017 and May 2017, we conducted a remote trial with a sample intervention at one nursing home site over 8 weeks. This trial explored the feasibility of remote data collection and transfer and the associated burden on participants in a novel setting. A falls prevention program acted as the intervention and was conducted by a private physiotherapy company, a third-party service provider. Their program involved weekly exercise classes and a daily exercise program over 6 weeks. The aim of the trial was not to assess the efficacy of this intervention—which was expected to be minimal in such a short period—rather, it was to explore the feasibility of remote data collection and transfer in a nursing home setting. Therefore, a number of technological solutions were selected for supporting activities of the trial (Table 1). These solutions were predominantly consumer devices, except for Quantitative Timed Up and Go (QTUG), a medical grade device for assessing fall risk. These solutions provided outcome measures for fall risk and mobility in older adults, offered a variety of data collection methods, offered an engagement tool, and were conducive to mobile data collection.

A participatory approach was used with the nursing home staff to codesign aspects of this study. Two focus groups were formed in the preliminary phase to explore current workflows and routines, existing assessment methods, and anticipated challenges associated with conducting a trial involving mobile and wearable technologies in this environment.

### Ethical Approval

Approval for the remote trial was granted the by University College Dublin Research Ethics Committee in February 2017.

**Table 1:** Mobile and wearable technology in a remote trial.

Technology	Description	Users	Data collection period
QTUG^a^ (Kinesis Health Technologies, Ireland) [24]	A sensor-based medical device that assesses gait, mobility, fall risk, and frailty while conducting a timed up and go performance test	Operated by physiotherapists and worn by residents	Pre- or postassessments conducted at weeks 1 or 8, respectively
Aging Research App (ICON Clinical Research, Ireland in partnership with mPROVE Health, US)	A tablet version of the Age-Related Muscle Loss Questionnaire adapted for self-assessing the impact of muscle loss on activities of daily living	Operated by residents and supported by the researcher	Conducted over weeks 2 and 3
Vívosmart HR (Garmin Ltd, US)	A wrist-worn watch that tracks daily activity, heart rate, and sleep patterns and acts as an engagement tool	Worn by residents, charged twice weekly by staff, and worn by 3× site administrators	Worn 24/7 from weeks 1-8
Galaxy J3 (Samsung, South Korea)	A mobile phone (smartphone) used for downloading data from Vívosmart HR and upload data to the cloud server	Operated by 3× site administrators	Worn 24/7 from weeks 1-8
Covalence (Big Cloud Analytics, US)	A platform for data analytics and visualization used for monitoring data collection and transfer	Operated by 3× site administrators and research team	Throughout the trial (from weeks 1 to 8)

^a^QTUG: Quantitative Timed Up and Go.

### Sampling and Recruitment

Using convenience sampling, we identified and enrolled a private nursing home in a middle-class suburb in Dublin. The nursing home had a population of 64 residents (as of February 24, 2017; see Table 2 for resident characteristics). A senior member of the nursing team was the first staff member enrolled in the trial. She was assigned the role of the trial co-ordinator (TC) and acted as a central point of contact between the research team and nursing home (see Table 3 for staff roles and responsibilities).

The TC and nursing home physiotherapist screened the resident population using the inclusion and exclusion criteria established by physiotherapists conducting the falls prevention program. Residents were eligible for inclusion if they exhibited (1) increased risk of falls (ie, experienced at least one fall in the last 6 months and/or scored moderate or high risk of falls in their last fall risk [FRASE] screening) and (2) were likely to benefit from taking part in a falls prevention program in the opinion of the TC or nursing home physiotherapist. Residents were excluded if (1) they were bed or chair bound; (2) required assistance of 2 people to walk; (3) had falls due to polypharmacy or unknown reasons; and (4) were clinically unstable according to TC; (5) were not likely to benefit from participating in the falls prevention program, for any reason, in the professional opinion of the TC or the nursing home physiotherapist (6) were fitted with a pacemaker.

The TC and nursing home physiotherapist identified 19 eligible residents (ie, 30% (19/64) of the nursing home population). The TC approached all eligible residents to invite them to meet the researchers. If they agreed, they were then brought to a private visitor’s room in the nursing home to meet 2 members of the research team (SD and BR) for an information session. The information session was piloted prior to being rolled out. BR took responsibility of assisting the TC in bringing residents to and from the room. SD explained the study to the residents using simple language. Researchers used the teach-back method for enhancing comprehension [24-27]. Visuals were used to indicate different components of the trial, featuring an image of the activity (eg, exercise class) and a picture of a person overseeing that activity (eg, the physiotherapists) as well as when and where the activity would take place. Physical devices, such as the Vivosmart HR device (hereafter referred to as the “watch”), were shown to the residents for them to touch and feel. Residents were provided with a summary and a long version of the participant information leaflets. They were given 5 days to review the material after which SD returned to the nursing home to answer any questions and collect written consents. In total, 11 residents were successfully enrolled in the trial (representing a response rate of 58%, 11/19). Of these residents, 5 provided independent consent and 6 provided consent by proxy. Reasons for nonparticipation varied from not being interested in the study to not taking part because fellow residents (ie, friends) were ineligible. Following resident recruitment, TC identified “reliable” health care assistants (HCA) ordinarily assigned to those residents. TC ensured that HCAs from each daytime shift were enrolled so that there was always a member of staff on hand to support trial activities. TC recruited two senior members of nursing staff as site administrators (SA) who were responsible for assisting with study activities and solving technical issues. Two physiotherapists from a private clinic were also enrolled to design and conduct the falls prevention program, and an activities co-ordinator (AC) was assigned for supporting the exercise classes.

In the first 2 weeks of the study, a member of the Big Cloud Analytics (BCA) team conducted 4 visits to the nursing home to provide education to residents on using the watch and to staff on charging the watch and using the mobile phone. They were also on site to set up the devices and address any problems. Prior to being rolled out, the educational process was piloted with an older female (aged 73 years) with low digital literacy. 

**Table 2:** Characteristics of residents.

Characteristic		Value	
Age (years), mean (SD)		80 (10)	
**Gender, n (%)**			
	Male		4 (36)
	Female		7 (64)
**Mobility**			
	Had a fall in past 6 months, n (%)		3 (27)
	Uses mobility aid, n (%)		4 (36)
	Timed up and go (seconds at baseline), mean (SD)		28 (17)
**MMSE^a^ score, n (%)**			
	30-25 (normal)		1 (9)
	24-21 (mild or early)		4 (36)
	20-10 (moderate)		2 (18)
	9-0 (severe)		4 (36)
**Exercise Class, n (%)**			
	Weekly attendance (out of 6 classes)		4 (79)

^a^MMSE: Mini-Mental State Examination.

**Table 3:** Staff recruitment, roles, and responsibilities in the remote trial.

Code	Role in the nursing home	Role in the trial	Responsibilities in the trial
TC (n=1)	Senior nursing staff	Trial co-ordinator and site administrator	Oversee trial activities daily Act as a central point of contact with the research team Recruit and enroll residents and staff Troubleshoot any technical issues
SA (n=2)	Nursing staff	Site administrator	Assist the trial co-ordinator with overseeing trial activities daily Troubleshoot any technical issues
HCA (n=6)	Health care assistant	Support	Charge the watch twice a week Assist residents with daily homework exercise program Assist residents with daily calendar entry
AC (n=1)	Activities co-ordinator	Support	Support the weekly falls prevention program by chaperoning residents to and from the exercise room Assist the Fit for Life physiotherapists when needed
Physiotherapists (n=2)	N/A^a^	Service provider	Design and conduct a fall prevention program with residents over 6 weeks (included weekly exercise classes and a daily homework exercise program) Conduct mobility assessments pre- or postfall prevention program (ie, QTUG^b^and static balance tests)

^a^N/A: not applicable.

^b^QTUG: Quantitative Timed Up and Go.

Residents were issued watches and shown how to use them to tell the time and count their steps. In terms of staff, the TC and SAs were provided with support manuals and trained to use the Covalence dashboard and how to access online support. HCAs were shown how to use the watch, complete a daily diary, and record the home work exercise program. Support manuals for staff were reviewed by the research team for clarity and accessibility before being issued to staff.

It should be noted that compensation—in terms of time or payment—was not offered to staff or residents taking part in the study. Although the research team and industry collaborators (Kinesis, ICON, and BCA) provided support to the study site, the trial predominantly relied upon existing care providers within the nursing home setting.

### Qualitative Research

A range of qualitative methods were embedded throughout the study to capture the lived experience of trial participants. During the 8-week trial, regular visits (1-2 times per week) were made by the research team (SD, OK, and BR) to conduct research activities, conduct observations, and troubleshoot on technical issues. Researchers also maintained regular phone and email contact. An issues log was kept by the research team throughout the trial to document interactions with staff relating to device troubleshooting. All participants were provided with a diary (described as an “experience calendar”) to complete daily or as often as they wished. It featured a calendar with stickers they could assign to any particular day to reflect the feelings they associated with participation. The stickers were adapted from an original faces scale [25]. These diaries were intended to act as a prompt during the semistructured interviews at the end of the trial. Results showing the residents’ activity data (heart rate, sleep patterns, and step count) and static balance were reviewed during the interviews.

Participants were then asked to consider the value of the trial in the context of the burdens they identified. At the end of the trial, semistructured interviews were conducted with staff (n=8) and residents (n=6). Staff interviews were conducted with 4 HCAs, 1 AC, 2 SAs, and the TC. Despite attempts, in some cases severe cognitive impairment and dementia meant resulted in 3 residents being unable to engage in exit interviews.

A team of experienced qualitative researchers, including a research assistant (OK), research lead (BR), and postdoctoral research fellow (SD) with backgrounds in anthropology, gerontology, and sociology, respectively, conducted the interviews. A topic guide was developed that included two distinct sections to explore the perceived burden and value of participation. When discussing burden, diaries (experience calendars) were used as a prompt for reflecting on their experience and assisting with recall. Once burden was captured, the interviewer showed the participant data generated by the activity tracker (heart rate, sleep patterns, and step count) as well as results of the static balance tests. These were provided on hard copy print outs that were given to the residents to keep. The participant was finally asked to consider the value of the trial in the context of burdens they had identified.

### Data Analysis

On average, interviews with staff lasted for 43 min, and interviews with residents lasted for 29 min. All interviews were digitally recorded and transcribed verbatim. Thematic analysis was conducted on these transcripts using NVivo 11 software package (QSR International Pty Ltd, Victoria, Australia) [28]. The analysis followed a largely grounded approach, with the exception of loosely imposed themes of “burden” and “value.” These themes provided an initial structure under which the interviews with residents were coded. First, by reviewing the resident interview transcripts, a comprehensive coding framework was generated. Analytical rigor was ensured using interrater and intrarater reliability testing. Samples of transcripts were coded by SD and OK, after which they were compared, reviewed, and discussed to resolve coding issues. Miles and Huberman recommend minimum interrater reliability levels of 0.70 and minimum intrarater reliability levels of 0.80 [29]. Using this standard, reliability levels were in the acceptable range, at 0.73 and 0.81 respectively.

For the staff interview transcripts, the framework generated from the resident interviews was duplicated and expanded with additional codes that were relevant to the staff experience (eg, time emerged as a burden for staff but not residents). As part of this iterative process, the staff codes were then synthesized with the resident codes. Additionally, team members as well as 2 expert colleagues from the center for Applied Research for Connected Health were consulted to review and provide feedback.

## Results

### Thematic Analysis

The thematic analysis is summarized in Table 4 and includes the number of sources (people) who quoted the respective theme and the number of mentions (quotes). Among staff, we found that the following eight characteristics of burden emerged: (1) comprehension, (2) time, (3) communication, (4) emotional load, (5) cognitive load, (6) research engagement, (7) logistical burden, and (8) product accountability. The watch emerged as a consistent source of burden throughout the trial for HCAs who were responsible for charging it. Similarly, the Samsung mobile phone (hereafter referred to as the “phone”) was a source of burden for SAs responsible for syncing it with the watch to support data transfer. Among residents, we found only four sources of burden: (1) comprehension, (2) adherence, (3) emotional load, and (4) personal space. Comprehension and emotional load were shared burdens across the two groups.

### Burden

#### Comprehension

Comprehension referred to the extent to which participants understood the research. Despite participants receiving detailed information about the study prior to providing informed consent, our results indicated that the trial was not consistently or fully understood by staff or residents at the nursing home. Among residents, there were various misinterpretations of the trial. One resident explained that she thought the daily exercises within the falls prevention program were being done “to keep your mind active” [Resident 5]. Other residents displayed an understanding of the purpose of the trial explaining that it was “preventing falls” [Resident 1]. However, the link between the watch and the intervention was unclear. When observing Resident 10 in conversation with another resident, she pointed to the watch and exclaimed, “How is a watch going to prevent falls?” [Resident 10]. However, *s* ome residents understood the basic function of the watch stating that “I understand [that it] was...counting steps…I think, to see how much we were walking. Is that it”? [Resident 9]*.*

**Table 4:** Codes applied, number of participants quoting the respective topic, and number of quotations within each theme.

Key themes				Participants, n		Quotes, n	
**Thematic analysis of nursing home staff (n=8)**							
	**Burden**						
		Comprehension	8		39		
		Time	7		57		
		Communication	7		43		
		Emotional load	7		34		
		Cognitive load	7		28		
		Research engagement	7		26		
		Logistical burden	6		20		
		Product accountability	5		7		
**Thematic analysis of nursing home residents (n=6)**							
	**Burden**						
		Comprehension	6		33		
		Adherence	5		7		
		Emotional load	1		1		

Residents’ cognitive impairments were potentially a factor which limited their understanding and imposed additional burden. At times, residents struggled to retain information about the research. When asked about her experience of taking part in the 8-week trial, a resident with mild cognitive impairment reported that “I don’t know because I don’t know what this thing is all about.” She also added, “What study? This study? Well I’ve only [learned] this for a few minutes, so I don’t know anything about it” [Resident 10].

Similarly, staff expressed difficulty understanding what the research was trying to achieve and what the devices were for as well as their roles and responsibilities within the trial. A senior nurse explained how her understanding shifted over time: “In the beginning, I understood that it was with regard to falls in the nursing home, but then I said that it has [little] to do with falls, it’s just for the technology to see how the technology works, I think”? [SA2]. HCAs’ understanding of the trial revolved around the watch—the device with which they had the most interaction. HCA 1 explained that “I knew it was obviously about reading the steps and the movement of residents, that’s what I understood [what] it was about. Just the kind of steps” [HCA1].

For staff, understanding their roles and responsibilities was also a burden. Staff in the dementia unit initially struggled with understanding how to use the experience calendar with their patients. HCA4 explains that although the study was explained to her:

I didn’t fully understand until you and your colleague [the researchers] came the following day or whatever day, until you explained fully. I didn’t get completely what I was supposed to do. Filling in their mood and stuff like that because they [dementia patients] couldn’t reply, they can’t tell me, you know that way? But, you then told me that I could answer for them. I was thinking “ah, this is never going to work,” if I was to ask them “are they OK with taking part?” because they have no understanding, you know?

HCA4

The burden associated with comprehending the role and tasks of site administration was evident in the comments of SA1. She expressed her frustrations:

It was a nightmare (laughs), no it was okay. So, when I started off, first probably through my own fault, I probably didn’t have enough of an insight as to what my role was. Kind of throughout the study I was kind of unsure what it was that I was meant to be doing. So, was I just meant to be overlooking the whole thing? Was it only meant to charge the watches? I was little bit unsure of what to do then with the [phone] in terms of how to sync it, but I asked questions, and I asked my colleague to show me.

SA1

She added that she “felt a bit stupid sometimes because I wanted to give you guys [research team] an answer but because I wasn’t sure [of the exact issue with the activity kits] myself…I felt a bit silly sometimes” [SA1]. Overall, the burden associated with comprehension—encompassing the trial, their roles, and the technology—was found to be the most pervasive aspect of burden in this study, as it emerged in some form in every interview.

#### Time

Staff reported lack of time to conduct research activities. Aside from the duration of tasks, time was constituted by many factors, for example disruption of workflow, interruption of tasks, and a lack of prioritization. Most notably, charging the watches was perceived as time-consuming. HCA5 explains that:

...It is time-consuming. By the time you go to the resident, get the watch off them, go up, plug it in…you get called on the way to do other things. And you have to put the residents’ needs before charging a watch, you know?

HCA5

HCA5 recognized that charging the watch was relatively quick but finding the time to do so was challenging: “Ah, it was easy enough to do [charge the watch]. It was just getting the time to do it. I know it sounds silly because it only takes like a minute or two.” [HCA5] The issue of having insufficient time was inherently explained in terms of being interrupted (ie, called to do other things) and prioritizing needs of the residents above the needs of the trial.

Completion of the daily homework exercise program with residents was also perceived to be burdensome. These exercises were part of the fall prevention program. HCA5 explained her lack of time to complete these exercises with residents: “We just don’t have time. We have other residents as well, upstairs we have 34 residents. And you just don’t have time to do the exercises with them every day.” [HCA5]

Overall, there was a lack of protected time for staff to complete trial-related tasks, and they simply had to add them to their current workload. When asked if she would take part in this type of research again, HCA4 replied:

…(Laughs) Not on top of my work, I wouldn’t, no. But, if you’re just doing like that, when you assign separate time to be able to…fully kind of focus on it, but no, I am just too busy in my work day.

HCA4

#### Communication

TC and two SAs provided local technical support to the research team. During the trial, communication between the nursing home and research team was largely one-way. The staff did not contact the research team for technical support at any point. However, the research team frequently contacted SAs. Continuous technical support was required throughout the trial to ensure data were transferred from the activity tracking kits to a remote server. BCA provided onsite tech support on two occasions between weeks 1 and 2 of the trial, and the research team provided technical support between weeks 3 and 6. In that time, 37 issues were logged with SAs at the nursing home and it took, on average, 4 days to resolve each issue. The communication and time burdens were enormously demanding on staff.

The trial appeared to increase the burden associated with communication between staff internally. Staff explained that when they would forget to charge the watch, they would need to contact one of their colleagues. HCA1 explained that:

I called one of the night staff to just tell one of the girls upstairs because it was one of the nurses downstairs that picked up. I asked her to tell one of the girls [upstairs] to put [the resident’s watch] in one of the chargers for me. The girls from the other team would know to take it out the next day, so it’s fine.

HCA1

In the interviews, staff reported difficulties with family members who did not understand the presence of the phone in the resident’s rooms. For example, HCA1 recalls one incident:

A family member came in for [Resident1] and I don’t know, well he didn’t really know that it was going on, but I presume it wasn’t him that they would have told about [the study]. So, he came in and was like ‘oh, what is this phone?’ as he thought it was a different phone. I said to him it was about the watch. It’s a case study that’s going on here and he was like “I didn’t know about it.” I was just like “oh my god, I’m so sorry about that” but somebody would have been told because the family has to know about it. So, it was just a bit of concern about the phone but I said it was for the watch and then that was about it, like there wasn’t much of a big deal about it, but he didn’t know about it.

HCA1

#### Emotional Load

Emotional load is based on the concept of “emotional labor” [30]. The term captures feelings of shame, embarrassment, and stress are often related to noncompletion of research tasks. In relation to charging the watch, one HCA remarked, “I was afraid to forget about them.” She explains, “…I [would] forget about the watches and I was always worried about [them]” [HCA6]. Another HCA reflected on the guilt she felt as a result of the watch not being charged and therefore not collecting data*,* “I felt bad because obviously it wasn’t recording anything for them…So it’s obviously going to have gaps here and there” [HCA4].

When discussing aspects of the trial that she deemed unsuccessful, TC attributed some personal blame:

The daily exercise thing was a massive flop, and I feel I am partly to blame for that. Because, um…I’d forgotten it. So, I know some were doing them and some weren’t, but I didn’t push on that.

TC

She explained her perception of staff feelings about the trial:

I did feel that they [staff] felt…like they’d kind of let the study down…because they just didn’t get around to doing [tasks]. I said look this is all learning, don’t worry if you don’t…get time to do it. Its fine, so don’t sweat about it…But I know there was a bit of that as well, you know?

TC

This sense of letting the study down was echoed by 1 resident. This resident had severe visual impairment and needed assistance of a staff member to fill out her experience calendar and complete her exercises. She explained how she was not inclined to interrupt the staff: “I hadn’t the courage to say would you do this with me when they were coming in for just about 10 minutes you know? So, I didn’t help you much there” [Resident 6]. This resident felt that she may have not been able to “help” data collection as a result.

#### Cognitive Load

Staff discussed the mental effort and burden associated with needing to remember to complete research tasks. This included charging the watch twice a week and assisting residents to complete daily exercises and their experience calendar. In this context, charging the watch was reported as particularly challenging. HCA4 reflected on the least enjoyable part of the trial as charging the watch, explaining that she was frustrated with “…trying to remember it, then forgetting it, and then remembering it” [HCA4]. Similarly, HCA1 also reflected on the burden associated with forgetting to charge the watch as the least enjoyable part of her experience stating that “Charging it (laughs). Charging it. Not that I don’t like [it], it’s just that I never remember to do it” [HCA1].

HCA1 outlined the context in which she was trying to complete the task of charging the watch. She explained that:

There were a few times when a good few hours would have past because I wouldn’t be getting them all at the same time to charge. It could be different, at the start [Resident 4, dementia patient], up until recently, would sleep until later. So, I wouldn’t be able to get his watch until later in the day to try charging, and maybe someone else would be earlier in the morning. Trying to remember was the thing, when you are busy.

HCA1

#### Research Engagement and Adherence

Although there was initial excitement and interest in the study, engaging in research activities over time became a burden for staff:

I didn’t mind it at the start…as the weeks went on I was getting a bit “ughh,” I have to keep doing it… then [I kept] forgetting the watches and then you’re like “ughh,” [if the watch is] obviously not on them…it’s not going to be recording [data] for you. And, just toward the end, I was like “I’m over it now” (laughs).

HCA4

Staff also perceived that residents disengaged with research activities over time and became nonadherent. HCA5 reflects that “At the start, for the first week or two, I did [Resident 5’s experience calendar] with her, but after that… I think some of the resident’s kind of lost interest in it, especially [Resident 5]” [HCA5]. Indeed, this resident reported that she had initially filled out her experience calendar but then stopped explaining, “I just never thought of it really” [Resident 5]. She reported that she also gradually forgot to complete the daily exercises, “I did it at first, with one of the girls [HCA]…After that, then I didn’t do it anymore…I didn’t think about it” [Resident 5]. Another resident explained that she did not engage with the experience calendar because she could not see the purpose of it:

I don’t think I did [fill it out]. I kind of said, “what's this for.” I pushed it aside and kind of said, “I think I'm jogging along reasonably well.” It was something…I kind of pushed aside. I paid no attention to it.

Resident 12

Another resident remarked that “I didn’t do it…because I didn’t know what I was doing” [Resident 1]. Residents who did perform the homework exercise program did not report any associated burden. However, a member of staff reported some discontent: “No they [the residents] never protest [about exercising], but there were sometimes they weren’t happy. They’d say ‘oh not again,’ but it depends on the day for everybody” [HCA6]. AC experienced some difficulty in motivating residents from the dementia unit to attend the weekly exercise class:

Some residents didn’t attend [the exercise class] particularly people with dementia. So one time [Resident 14] …and I don’t know if he remembered…when I said “we go for exercises,” he didn’t want to go, but I think it’s kind of dementia you know.

AC

#### Logistical Burden

The nursing home contained 4 wards spread over 2 floors. Mobile phones and chargers for watches were set up in the participating resident’s bedrooms. Residents were recruited from every ward; therefore, their bedrooms and the respective devices and equipment were dispersed across every ward. It appears that this presented a logistical burden for SAs who needed to move among different locations within the nursing home to check and sync devices. This was regarded as highly inconvenient:

You need to go take the watch to the phone, take the phone to the watch, person to the phone…when it wasn’t syncing. It took a long time to get through them. And it was never just one, there was always more than one, you know if it was one, it takes 5 minutes, but…there was upstairs, downstairs. Residents are here, residents are in the dining room.

TC

Each watch was identical, but had a unique identifying code (UIC) placed on its underside. As is standard practice in research, for data protection purposes staff, did not have the key between the UIC and the names of the residents. However, this created a burden for staff when attempting to collect and charge multiple watches at once. HCA6 explains “I never took all of [the watches] together. I only took them one-by-one…I never wanted to mix them up” [HCA6]*.* She explains*:*

I think one time I took two…and I said left (hand) is [Resident 9] and the right one is [Resident 10] (laughs). Or I look at the number on the code and say okay, this is (910) or (374). So, (910) is [Resident 10] and (374) is [Resident 9].

HCA6

#### Product Accountability

There was a burden associated with responsibility to keep the devices safe and secure so that they were not damaged or broken. Staff appeared to be conscious of the cost of the devices. In their supervisory role as SAs, senior nurses took it upon themselves to ensure that the activity trackers issued to residents throughout the nursing home were kept safe. SA1 was conscious that these products were not their property and was apologetic in instances where they were damaged:

In terms of the residents, I would always be keeping an eye; there were two watches, as you know, that were a little bit damaged by the residents for whatever reasons. I did say to [the researcher] that they were damaged and I apologized for that but, in general, we, the staff, would make sure that they were being looked after and weren’t thrown around. So, yeah we would be conscious as they weren’t our property.

SA1

The phones were often placed on the floor under the resident’s nightstand to keep them out of the way. SA2 reported awareness that the products may become damaged by routine cleaning:

I was more aware of the cleaning staff because some of the phones were on the floor, so I was more aware not to wet them or not to damage them in that way. But, the HCA were very careful and I knew they were going to take care of it, just cleaning staff more so than anyone else. I made sure they were on the skirting boards and not on the actual floor.

SA2

One staff member reported an incident where a phone went missing:

One day, one of the families unplugged the whole phone and everything because they thought it was a staff member charging their phone. Even though they had been told about the study, they just didn’t want them charged in the room. Then, I had to go, plug it all back in, and make sure it was working.

HCA5

This HCA described this encounter with the family member as “awkward.” Locating the device was challenging and stressful for HCA: “I had to go and find it because her daughter had put it away, and then [the resident] had moved it…at first, I thought part of it was missing and I was like ‘Oh God, where did it go?’” At this point, she described an emotional burden related to her responsibility and accountability for the device. She reported feeling panicked and thinking “God, if it is gone, what are we gonna do?” [HCA5]. However, the phone was ultimately found and the presence of the device was re-explained to the family member. HCA felt this resolved the issue.

#### Personal Space

Conducting research in a residential setting has the potential of reduce privacy for residents in their home. During the 8 weeks of the trial, their phones were frequently checked by SAs and the researcher in the resident’s rooms. Sometimes, this happened when the resident was not in his or her room and may have created unease if the resident thought someone had been in their personal space. One resident explained:

Funny I thought that someone was mooching around my clothes, affairs, and things like that now…don’t know whether I lost anything or not…I thought that someone was coming into the place.

Resident 12

### Relationship Between Burden and Value

While participants reported to find value in data, this was found to be somewhat superficial. For example, one member of staff reported that “I just find it interesting, I don’t know [why]” [HCA6]. Value was largely expressed in terms of its absence rather than its presence. Staff struggled to understand how tracking heart rate, step count, and sleep patterns could be useful to them in their everyday life and how it related to preventing falls. One staff member explained “I think it’s useful (dubious tone). Yeah, no I do, but I would have to look at it a little bit more just to see how this can help us to further prevent falls” [SA1]. Similarly, abstract relationship between the watch and falls preventions was a barrier for residents. When the interviewer explained that the watch was counting steps, one resident exclaimed, “What the hell difference does that make? Sorry for my language” [Resident 10].

TC felt they captured much of this data informally anyway and would communicate it in handover meetings between day and night shifts:

Generally, our communication systems around our hand over are that…those type of things are our main highlighters. In terms of a hand over, [it] would be, “Did they sleep well?” “Did they have any falls today?” “Did they eat?” “Did they eat well?” “Are they drinking well?” All that type of thing is basic. [So we are] doing that anyway.

TC

The perceived value of data may have been comprised by skepticism over the integrity of data collection. A staff member reflects:

Well I don’t think you got the full information that you wanted. Did you get the full information that you wanted?I’m still not sure if I helped you enough to have your study fully, fully completed.

SA2

TC explained, “I don’t know the relevance of it. Is it going to be, will it be accurate? You know, in terms of feedback, just because there are so many gaps and holes in it” [TC]. Overall, analysis suggested that the value of data appeared to be limited for participants, relative to the burden they encountered.

## Discussion

### Principal Findings

Mobile and wearable technologies offer the potential to conduct clinical trials remotely in a nonclinical setting, thereby reducing the associated burden on patients. However, this study suggests that the burden associated with the remote trial was not modulated by the value of participation. Staff burden emerged to be multifaceted and interrelated in practice. For example, a cognitive burden (ie, forgetting to charge the watch), may have prompted an emotional burden (ie, anxiety about the battery dying) and posed a logistical burden (ie, phoning a colleague on the ground floor to instruct a colleague on the first floor to charge the watch). Thus, burden should be considered as multifaceted in practice.

Comprehension emerged as the most commonly shared burden among residents and staff in the trial. Inability to comprehend the purpose of the trial—particularly the relationship between the intervention and wearable device—was a substantial challenge and may have contributed to a lack of motivation and engagement to participate in trial activities. Thus, the need for participants to understand and “buy in” to the concept of the study is arguably paramount [31]. Indeed, there is a large amount of evidence to suggest that comprehension can pose a barrier to clinical trial participation and recruitment [32-34]. Our findings suggest this is also relevant in a remote trial where the relationship between the intervention and devices used for supporting the intervention have the potential to be unclear.

### Staff Experiences

A lack of adherence among staff is a recognized barrier to research in nursing homes [35]. Nonadherence among staff in this study may have been related to the experience of burden. Thus, it is useful to further analyze adherence to better understand how it is constituted in this setting.

### Communication and Feedback

Communication was the most frequently cited burden. Although the research team made regular site visits, the informal nature of capturing feedback was not sufficient. The participatory approach enabling feedback and dialog between the staff and research team was front-loaded into the study design. However, communication may have been improved by formalizing a feedback loop with staff, residents, and their families throughout the trial. This could have enabled the research team to accurately capture and respond to emerging issues, thereby improving the experience for all stakeholders. For example, having a staff member assigned to the collection and charging of devices in the evening and another assigned to the return of devices each morning may have been more effective and may have created less logistical concerns. This would require careful labeling of devices to ensure consistency of use.

### Workflow

Although the study protocol was developed with nursing home staff input, embedding tasks and activities into the work plans of staff might have helped in alleviating some communication and role or responsibility issues. Ensuring that study procedures are integrated into daily activities and task list of the care staff is important. Remuneration for the nursing home staff, as would be the case in a typical clinical trial, may have helped in achieving this. While the protocol was developed with staff input, protected time for TC and senior nursing staff to embed tasks and activities into work schedules might have helped in alleviating some communication and role or responsibility issues. Similarly, protected time for staff to execute these activities might also have reduced burden. Although efforts were made to build a collaborative design, there are known structural and cultural issues around the execution of participatory approaches that are not unique to nursing home settings [36-38]. Arguably, a more comprehensive, rather than a piecemeal approach to participatory research is required.

The job of integrating research activities into the routine workflow of the nursing home was the responsibility of TC. Although her knowledge of staff shifts and vast experience in the nursing home meant that she was best placed to oversee this, a lack of protected time to devote to her role and to train and work with the research team was challenging. Her responsibilities also shifted over time. For example, it was intended that TC would be responsible for recruitment of residents. However, in practice, managing this task alongside her senior role in the nursing home was not feasible. It is noteworthy that AC was the only staff member who did not identify time as a burden. She was not assigned with any tasks that fell outside of her typical workflow (ie, assisting completion of the daily exercise program or experience calendar with residents or with charging the watch).

### Exposure to Value

In this study, we explored the burden associated with a remote trial, and this study was not designed to explicitly measure values. Therefore, there were limited opportunities for participants to become exposed to data. It was assumed that there would be inherent value in participation in the form of resident’s receiving data from the watch and that this would provide insight to staff. However, a report of the full clinical data (ie, step count, sleep patterns, and heart rate) was only shared with participants at the *end* of the study. Thus, staff and residents only had the opportunity to become exposed to the value of the data at the end of the trial, once the burden associated with participation was over. The only opportunity for feedback of data during the trial was to the residents in the form of the step count captured by the watch. Yet with the exception of one resident, it appears that the residents generally did not interact with the watch. Arguably, the conception of the trial was overly abstract and contributed to a lack of comprehension of the trial and study. The lack of exposure to value is problematic in that it is therefore unable to modulate the perceived burden and may indeed inflate the perception of burden.

### Connectivity

For staff, burden was likely amplified by persistent problems with connectivity at the research site. This resulted in the research team frequently phoning and emailing the SAs to conduct localized troubleshooting. Arguably, this increased burden and restricted the potential value of the trial. This may have been compounded by a lack of protected time for the staff at the nursing home to conduct research activities and troubleshooting.

### Residents’ Experiences

Resident burden was found to be relatively limited. Where it was observed, it was mainly related to comprehension and adherence, with some evidence of burden relating to emotional load and personal space. There also appeared to be interdependency between comprehension and adherence, whereby a resident may not have understood why he or she needed to complete a research activity. Therefore, many residents did not engage with the research. Data security did not emerge as an issue in interviews with residents. This issue was also not raised by staff or families during the trial.

### Engagement

It was assumed that the watch, which was a consumer device, would act as an engaging tool for residents (and staff), potentially acting as a talking point and generating competition among users. However, it was observed by staff and the research team that only one resident engaged with the watch as a step counter. Among this cohort, there is no evidence to suggest that inclusion of mobile and wearable technology improved participation, engagement, retention, or adherence. However, convenience of the remote trial did allow for the inclusion of typically hard-to-reach individuals, such as patients with dementia.

### Limitations

#### Specificity of Site and Devices

The findings of this study refer to a remote trial conducted in a specific nursing home setting using specific health technology devices. This research was conducted at a single site; therefore, the extent to which unique aspects of this one setting—within this one organizational culture—contributed to the specific findings is unknown. The burden associated with technology for health care staff, however, is not necessarily unique to this setting. The problem of “real-world” deployment is recognized within the literature, particularly in terms of workflow integration and conceptualization [39,40]. Indeed, the evidence base to support clinical claims of eHealth technology has been debated, and it has been suggested that costs associated with commonly deployed eHealth technologies (ie, time and infrastructure) may in fact decrease organizational efficiency [39].

Thus, before we can overcome these burdens, qualitative exploratory studies—of the kind presented here—are crucial for understanding how and why burden manifests. The devices used in this study were chosen because they were widely available to the research team, which had worked with this technology in previous studies. It is possible that the choice of devices may have contributed to some of the burdens identified in this study, although the extent of this burden is unknown. Although comparing a range of devices and their limitations was beyond the scope of this study, this should be considered in future research.

#### Information Overload

In terms of comprehension, it is possible that in our efforts to be transparent and didactic during recruitment, we risked overloading the residents with information. Although there is no evidence to suggest that this interfered with their willingness to enroll in the study or their ability to retain information over the 8-week period, effectiveness of teach-back methods for information retention over time (ie, a clinical trial period) requires further study [41-43].

#### Opportunities for Feedback

As part of a participatory approach, focus group studies were conducted with staff prior to the trial being designed. However, this was not continued throughout the trial. Formalizing a feedback loop may have improved communication and eased the burden associated with comprehension. Although regular visits were made by the research team to the nursing home, whereby feedback was captured informally, more structured follow-ups with staff and residents may have enabled the research team to determine how much information was actually being retained, address issue or gaps in knowledge in a timely manner, and reinforce the purpose of the study.

#### Staff Training and Protected Time

This study involved leveraging the resource of research-naïve health care professionals. Increasing training resources available at the start of and during the study may have been useful in alleviating some of the issues identified. In particular, comprehensive face-to-face training, which specifically addresses aims, objectives, and importance of the study, should be conducted for all study staff. Indeed, the need for more formalized staff training around the implementation of connected health technologies has been found outside of the nursing home setting, for example in community settings [38].

Similar to that observed with many clinical trials, finding approaches for effectively training personnel who are not able to attend face-to-face trainings because of operational commitments, shifts, or working patterns is an important consideration. A possible solution could be online or app-based study guides to provide more convenient and immediate methods to access training and troubleshooting materials. Progress feedback reports during the study identifying progress to date and detailing issues encountered and how to resolve them would provide an additional method to re-iterate study objectives and importance. Again, formalized meetings with staff should also be in place to support dialog between staff and researchers.

Staff were not remunerated for the additional work required by the study. Remuneration could have provided staff with protected time to engage in training and meetings. This in turn, may have improved communication during the trial. It is also possible that remuneration for the nursing home staff may have provided additional motivation.

In addition to the operation of the study, training should include defining roles and responsibilities. Clearer definition and communication of the roles and responsibilities of staff may have reduced the burden associated with comprehension. Again, this would have been better facilitated by ensuring protected time for staff.

### Conclusions

The paper identifies and characterizes perceived burden associated with a remote trial in a particularly challenging patient population. It identifies eight aspects of staff burden and four aspects of resident burden that could be further explored and developed. The exploratory nature of this research meant that there were many unknowns before entering the research site. From the outset, we assumed that the staff burden associated with a remote trial would be related to increased workload and the time consuming nature of co-ordination. Although these burdens were indeed observed in the staff experience, their nuance, variation, and extent were relatively unexplored until now. This research, therefore, offers a novel understanding of the nature of staff burden in a remote trial and underlines the importance of the relationship between burden and value. The potential of remote clinical trials requires further examination to optimize and enhance the methodology. This study suggests the convenience of a remote trial that can aid inclusion of hard-to-reach patient groups; however, we need to comprehensively measure and minimize the associated burden on relevant stakeholders.

## Abbreviations

AC: activities co-ordinator
BCA: Big Cloud Analytics, Inc
HCA: health care assistant
MMSE: Mini-Mental State Examination
QTUG: Quantitative Timed Up and Go
SA: site administrator
TC: trial co-ordinator
